# Radiation pneumonitis and pulmonary function with lung dose–volume constraints in breast cancer irradiation

**DOI:** 10.1017/S1460396913000228

**Published:** 2013-06-07

**Authors:** U. Blom Goldman, M. Anderson, B. Wennberg, P. Lind

**Affiliations:** 1 Department of Oncology, Karolinska University Hospital, Stockholm, Sweden; 2 Department of Oncology-Pathology, Karolinska Institutet, Stockholm, Sweden; 3 Department of Physiology, Stockholm Söder Hospital, Stockholm, Sweden; 4 Department of Medical Physics, Karolinska University Hospital, Stockholm, Sweden; 5 Department of Oncology Sörmland, Mälarsjukhuset, Eskilstuna, Sweden

**Keywords:** breast cancer, dose–volume histogram, loco-regional radiotherapy, pulmonary function, radiation pneumonitis, radiotherapy

## Abstract

**Purpose:**

We studied symptomatic radiation pneumonitis (RP) and changes in pulmonary function tests (PFTs) after loco-regional radiotherapy (LRRT) with *V*_20_ lung constraints in breast cancer (BC).

**Patients and methods:**

Sixty-four women underwent PFTs before and 5 months after 3D planned LRRT for BC. The incidentally irradiated ipsilateral lung *V*_20_ was minimised to <30%. Patients were monitored for symptoms of RP 1, 4 and 7 months after radiotherapy (RT) and data on covariates were collected prospectively. The outcome was compared with previous treatment series.

**Results:**

Pneumonitis was less frequent with the applied constraint, that is, four mild and one moderate case, than in our previous report (*p* < 0·001). In multivariate analyses, neither dosimetric data nor covariates appeared to influence mean changes in vital capacity [−0·11L, standard error of the mean (SEM) 0·03] or diffusing capacity of the lung for carbon monoxide (DLCO) (−0·20 mmol/kPa/min, SEM 0·01), except for pre-RT chemotherapy, which diminished the change in DLCO 5 months post-RT.

**Conclusions:**

The used constraint and 3D planning lowered the rate of RP and short-term changes in PFTs compared with our previous treatment series. Pre-RT chemotherapy affects DLCO baseline levels. Rates of side effects should be continuously studied when new target definitions or therapies are introduced in LRRT of BC.

## Introduction

Radiotherapy (RT) is the most effective method to eradicate the remaining diseases after breast cancer (BC) surgery. The meta-analyses by the Early Breast Cancer Trialists’ Collaborative Group demonstrate a significant benefit in both BC-specific and overall survival after 15 years, following postoperative RT.^1^ ^,^ ^2^ However, RT is also known to cause early and late side effects in surrounding tissues, for example, heart and lung.

The lung is sensitive to ionising radiation and side effects may arise, as acute pneumonitis and late lung fibrosis. The risk for acute and chronic RT-induced lung morbidity is influenced by irradiated lung volume, total dose and dose per fraction.^3^ ^,^ ^4^ Clinically, significant symptomatic radiation pneumonitis (RP) occurs in 1–10% of patients irradiated for BC with modern RT techniques.^5^ The addition of regional nodal irradiation to breast RT significantly increases the treatment volume and the incidence of side effects.^6^ With today's 3D RT-planning techniques, we can individually quantify and limit the amount of incidentally irradiated lung volume. Clinical data suggest that a total lung dose of more than 20 Gy given with conventional fractionation should be avoided if the unirradiated lung volume is not sufficient to guarantee essential breathing function.^7^ In our previous trial,^8^ we investigated short-term pulmonary side effects in BC patients after adjuvant RT. No case of RP was found in patients who received doses of ≥20 Gy to <30% of the ipsilateral lung volume, that is, *V*_20_ < 30.^8^ We therefore used this cut-off level in the present trial, and this is also the quantitative analysis of normal tissue effects in clinic (QUANTEC) recommendation.^9^

Other study groups have found relations between chemotherapy and tamoxifen intake and RT-induced lung toxicity.^10^ ^,^ ^11^ In one of our previous studies, we found an association also with age and post-RT changes on chest X-ray.^12^

Individual biological factors can be important for the sensitivity to irradiation. Thus, possession of some gene variants can predict the development of enhanced adverse effects after RT, for example, the ATM gene.^13^ In contrast, smoking has been reported to reduce the risk of RP by suppression of the local inflammatory reaction.^14^ ^,^ ^15^

We have previously studied short-term pulmonary function loss in irradiatied BC patients by pulmonary function test (PFTs), 5 months after RT. A clinically significant reduction of PFT's function was observed among patients treated with loco-regional radiotherapy (LRRT) who developed symptomatic RP.^16^

The present study investigated short-term RP and changes in PFTs in LRRT when an ipsilateral lung dose–volume constraints of *V*_20_ <30% was applied and the results were compared with our two previous reports.^8^ ^,^ ^16^

## Patients and methods

This is a prospective study on RP and changes in PFTs after 3D planning with lung dose–volume constraints but without sacrifice of adequate coverage of the target volume (TV). The local ethics committee approved this trial. Furthermore, participating women gave informed consent before study enrolment.

### Study population

All women who were referred for adjuvant LRRT after surgery for node-positive BC from November 2002 to March 2005 were asked to participate in this trial. A total of 91 patients were included in the study, but two patients withdrew their consent owing to early relapse. Of the patients, 89 patients were followed for 7 months after RT for symptoms of acute/subacute RP. Twenty-five patients declined to undergo the post-RT PFTs. The remaining 64 women were examined with PFTs before and 5 months after RT. Of the patients, 69 women had undergone mastectomy and 22 had undergone lumpectomy. Seventy-four patients were irradiated with LRRT to the chest wall or breast, axilla and supraclavicular region and in these patients the internal mammary lymph nodes (IMN) were included. Ten patients received LRRT, excluding the IMN. Seven patients were referred for RT to the axilla and supraclavicular fossa only.

The mean age was 55 years (range: 32–81). Data on potential confounding covariates were collected prospectively, that is, history of cardiovascular or pulmonary co-morbidity, smoking habits, functional level (i.e., not being able to climb three flights of stairs without a rest due to shortness of breath) and adjuvant hormonal, trastuzumab and chemotherapy treatment. The study population had no major medical conditions and had good functional levels at baseline. The chemotherapy was concluded 3–4 weeks before RT. Concurrent chemotherapy was never given. The most common regime consisted of FEC (5-fluorouracil 600 mg/m^2^, epirubicin 60–75 mg/m^2^ and cyclophosphamide 600 mg/m^2^/dL q 3 weeks × 6). In 11 patients, the therapy included docetaxel 75 mg/m^2^. Six patients received trastuzumab during RT. Intake of tamoxifen and anastrozol during RT was evenly split among the women, 24 versus 26.

### RT treatment techniques

All patients were placed in an identical supine fixed position, with the arms elevated above the head, during the CT session, simulation and treatment. The used RT techniques are described in detail in an earlier publication.^16^

#### LRRT after modified radical mastectomy

For LRRT following modified radical mastectomy, the target was defined as the chest wall, the upper IMN, axillary and supraclavicular lymph nodes. The treatment was delivered with one anterior electron beam (range: 6–12 MeV) covering the chest wall and the IMN. The energy was selected so that the 95% isodose covered one-half of the rib thickness of the chest wall. The supraclavicular fossa and axilla was covered with a 6 MV photon beam. The dose was prescribed at 3 cm depth. A small posterior photon beam (8 MV) was added for the axilla and the 2 Gy dose was prescribed at its centre. A total dose of 46 Gy with 2 Gy/day, five fractions/week, was used.

#### LRRT after partial mastectomy without the IMN

The breast parenchyma was treated with two tangential photon beams (4 or 6 MV) and the regional lymph nodes were irradiated in a similar way as described above. The dose to the lymph nodes was 46 Gy and the dose to breast parenchyma was 50 Gy with a fractionation of 2 Gy/day, five fractions/week.

### LRRT after partial mastectomy including the IMN

An oblique electron beam was added to include the upper IMN in the TV.

### Treatment planning and dose–volume histogram

CT-based 3D treatment planning (Pinnacle; version 6.2b) was performed for each patient.

Treatment planning aimed at good coverage of the TV and conformed to the ICRU norms and at avoiding a dose in excess of 20 Gy to more than 30% of the ipsilateral lung volume. The cumulative dose–volume histograms were calculated and the ipsilateral lung volume receiving ≥13 Gy (*V*_13_), ≥20 Gy (*V*_20_), ≥30 Gy (*V*_30_) and the mean lung dose (MLD) were recorded.

### Monitoring of symptomatic RP

The patients were followed 1, 4 and 7 months after RT for respiratory symptoms, that is, cough, dyspnoea with or without fever. All patients were classified into three groups according to the CTC criteria (version 2.0).

Grade 0. No respiratory symptoms.

Grade 1. Mild: cough and/or dyspnoea with or without fever judged to be radiation induced.

Grade 2. Moderate: same as 1 but with impaired daily functions and treated with corticosteroids.

Chest X-ray was recommended as a diagnostic in patients with respiratory symptoms.

A standardised chest X-ray was also performed after 5 months in all patients.

### PFTs

PFTs were conducted before and after 5 months of completion of RT. Body plethysmography was used to measure vital capacity (VC), and the mean of three adequate measurements was reported. Diffusion capacity of carbon monoxide (DLCO) of the lung was determined by the single breath CO method and corrected for the haemoglobin levels. The better of the two acceptable measurements was used. The same physiology laboratory was used for the PFTs in both the present and our previous trial.

### Statistical analysis

The Student *t*-test was used to analyse differences in the mean *V*_20_ and *V*_30_ in the previous and present trials. The *χ* ^2^ trend test was used to test differences in RP rates between the previous and present trials. Multiple linear regressions were used to analyse changes in VC and DLCO in relation to the above-mentioned dosimetric factors and the above listed potential confounders. All test were two-sided, and *p*-values of <0·05 were considered statistically significant.

## Results

**Figure 1:**
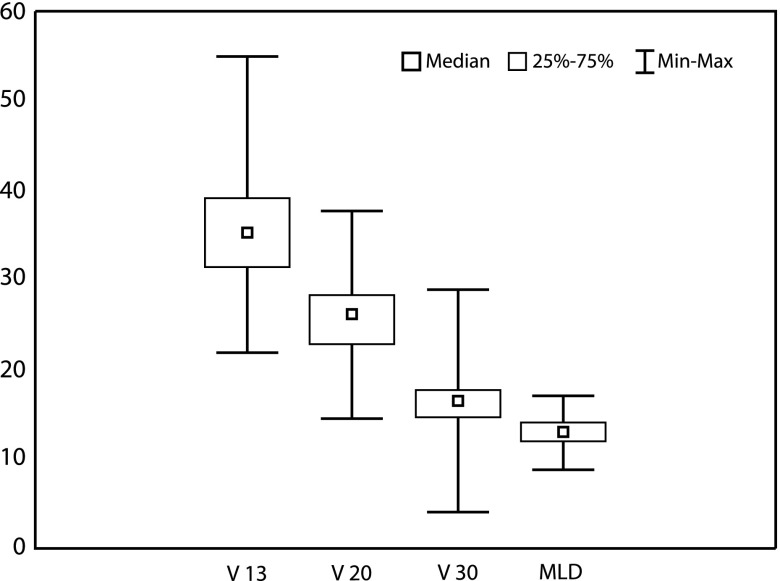
Distribution of ipsilateral lung dose–volume dosimetric data in present trial for loco-regional radiotherapy techniques, including the internal mammary lymph nodes.

**Figure 2:**
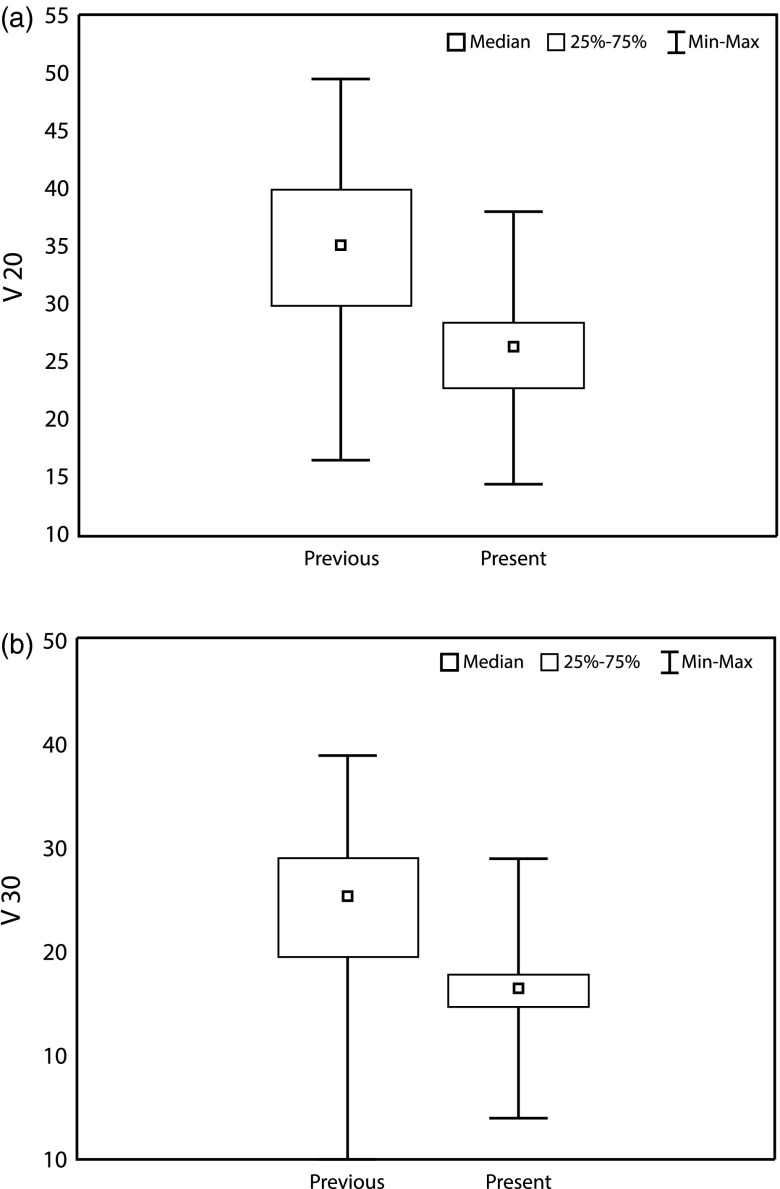
Distribution of ipsilateral lung (a) V_20_ for loco-regional radiotherapy techniques, including the internal mammary lymph nodes in previous (mean 35%) versus present (mean 26%) trial; (b) V_30_ for loco-regional radiotherapy techniques, including the internal mammary lymph nodes in previous (mean 24%) versus present (mean 16%) trial.

The distribution of the lung dosimetric data *V*_13_, *V*_20_, *V*_30_ and MLD for LRRT including the IMN with ipsilateral *V*_20_ constraints in the present trial is presented in Figure 1. Our intent was to adhere to the *V*_20_ lung-dose constraint of ≤30%, but in a few cases we had to accept a somewhat higher lung dose because of the nature of the patient's anatomy. There was a statistical significant reduction in mean *V*_20_ (35% versus 26%) and *V*_30_ (24% versus 16%) when comparing the present with previous trial for RT techniques, including the IMN (Figure 2a and 2b).

RP was rare with the applied ipsilateral lung dose–volume constraint *V*_20_ ≤ 30% (Table 1). Mild RP was detected in four patients, and one patient developed moderate RP and was thus treated with corticosteroids. There was no severe reaction (Grade 3–4) in this study (*n* = 89). In comparison, cases of mild and moderate RP was more frequent in our previous report (*p* < 0·001) (Table 1). When we reanalysed the post-RT PFT changes in our previous trial (*n* = 217) for relations with individual dosimetric data, that is, *V*_20_ and *V*_30_, and tested the effect of potential confounding factors by multivariate analysis (MVA), that is, tamoxifen use, chemotherapy, smoking habits and age, we found associations between the factors *V*_20_ and *V*_30_ and loss of VC (*V*_20_ *p* < 0·001 and *V*_30_ *p* < 0·001) and DLCO (*V*_20_ *p* = 0·05 and *V*_30_ *p* = 0·02). Furthermore, tamoxifen intake during RT appeared to increase the VC changes (MVA *V*_20_; *p* = 0·005 and MVA *V*_30_; *p* = 0·002). Pre-RT chemotherapy diminished the change in DLCO, 5 months post-RT in both the *V*_20_ and *V*_30_ MVAs, as patients having undergone pre-RT chemotherapy had lower baseline values, because of lung toxicity of the used drugs that appeared to partly normalise 5 months post-RT.

**Table 1:** RP in the previous (n = 217) and present (n = 89) trials with LRRT techniques, including the IMN

RP grade	None (G0)	Mild (G1)	Moderate (G2)
Previous study	149	43	25
Present study	84	4	1

*Note*: *χ* ^2^ trend test *p* < 0·001.

*Abbreviations*: RP, radiation pneumonitis; LRRT, loco-regional radiotherapy; IMN, internal mammary lymph nodes.

In MVA of the present trial, neither dosimetric data nor covariates appeared to influence the observed mean changes in VC (−0·11 L, SEM 0·03, *p* = 0·007) or DLCO (−0·20 mmol/kPa min, SEM 0·01, *p* = 0·01) 5 months post-RT, except for the above-mentioned effect of pre-RT chemotherapy, which again was associated with less DLCO decrease. Furthermore, the mean changes in VC and DLCO appeared lower than in our previous report in which a constraint was not used, that is, −0·15 L and −0·39 mmol/kPa/min.^16^

## Discussion

When we applied the ipsilateral lung volume constraint of *V*_20_ ≤ 30% in our 3D planning of LRRT in BC, symptomatic RP was rare and less frequent than in our previous trial. We found no correlation between the dosimetric factors nor covariates and PFTs changes, that is, DLCO and VC, in the present trial except for pre-RT chemotherapy and less post-RT DLCO changes. This observation was probably because of lower baseline values in patients receiving chemotherapy. However, dosimetric data were associated with reductions in PFTs in our previous treatment series. The lack of relation between dosimetric factors and decline in PFTs in the present trial may be due to study size and the observed small mean changes in VC and DLCO with the used constraint. DLCO is one of the most sensitive variables for pulmonary function changes due to drug-induced toxicity.^17^ Chemotherapy was always completed 3–4 weeks before RT in both trials. The most common chemotherapy regimes included in the previous trial was CMF (600 mg/m^2^ cyclophoshamide, 40 mg/m^2^ methotrexate and 600 mg/m^2^ 5-FU). Both cyclophoshamide and methotrexate are known to cause pulmonary toxicity by local inflammation in the lung parenchyma and this may affect the gas exchange.^17^ Eighty per cent of the women in the present trial received chemotherapy and the most common treatment was the FEC combination. A few patients also received taxanes. Other investigators have reported an increased risk of RP when chemotherapy, including paclitaxel, was administrated concurrently or sequentially with RT.^18^

Ten per cent of the women in the previous trial^16^ experienced moderate RP and needed corticosteroid treatment. The mean reduction in VC in the latter group was equivalent to 15 years of normal ageing or loss of three-fourth lung lobe.^16^ Decrease of parenchyma elasticity in the irradiated part of the lung is suggested to inflict the reduction of VC.

Some reports suggest that tamoxifen influences the risk for post-RT fibrosis, but other studies have failed to detect this effect.^10^ ^,^ ^19^ We have previously reported that concomitant tamoxifen has no influence on VC and DLCO;^16^ however, when reanalysed, women treated with LRRT, including the IMN in our earlier trial and included individual dosimetric data, we found a possible relation with VC changes. Today, however, the use of aromatase inhibitors is more frequent in postmenopausal women. The CO–HO–RT trial demonstrated that it appears safe to use an aromatase inhibitor during RT with respect to early side effects, but the long-term effects are not yet evaluated.^20^ We did not detect any deterioration of PFTs among the few number of patients receiving trastuzumab concomitantly with RT in the present trial. Pneumonitis in sequentially administrated trastuzumab is rarely seen.^21^ ^,^ ^22^

The need for irradiation of the IMN for patients with 1–3 node-positive BC is still under debate, and many centres have excluded radiation to the lower IMN. However, Whelan et al.^23^ reported at ASCO 2011 a benefit in the MA20 trial also for this group in terms of reduced loco-regional recurrence. The RTOG has published guidelines for post-mastectomy LRRT.^23^ If we strictly follow their suggested TVs, the per cent ipsilateral lung *V*_20_ would be considerably higher than in our present report,^24^ and this could probably lead to a higher RP rate.

Other groups have evaluated patients for changes in PFTs for 3–10 years and have proposed that lung function changes may follow a biphasic pattern with a partial recovery after 12 months, followed by a late progressive worsening after 8–10 years.^25^ The reduction was seen in total lung capacity and DLCO and in patients receiving concomitant tamoxifen. There are also reports on increased risk for secondary lung cancer on the irradiated side, especially in smokers,^26^ which further strengthens our goal of minimising the incidentally irradiated lung volume.

In conclusion, with 3D RT-planning and an ipsilateral lung dose–volume constraint of *V*_20_ ≤ 30%, we have reduced the rates of RP and changes in short-term pulmonary function at our department. Thus, individual 3D dose planning and lung–dose–volume histogram analysis is of importance in LRRT of BC. Ideally, baseline PFTs should be acquired before start of all therapies, as chemotherapy affects the diffusion capacity of lung. Rates of side effects should be continuously followed when altered target definitions or therapies are introduced in LRRT of BC.

## References

[1] Darby S, McGale P, Correa C et al Effect of radiotherapy after breast-conserving surgery on 10-year recurrence and 15-year breast cancer death: meta- analysis of individual patient data for 10,801 women in 17 randomised trials. Lancet 2011; 378 (9804): 1707–1716 22019144 10.1016/S0140-6736(11)61629-2 PMC3254252

[2] Clarke M, Collins R, Darby S et al Effects of radiotherapy and of differences in the extent of surgery for early breast cancer on local recurrence and 15-year survival: an overview of the randomised trials. Lancet 2005; 366 (9503): 2087–2106 16360786 10.1016/S0140-6736(05)67887-7

[3] Overgaard M, Bentzen S M, Christensen J J, Madsen E H The value of the NSD formula in equation of acute and late radiation complications in normal tissue following 2 and 5 fractions per week in breast cancer patients treated with postmastectomy irradiation. Radiother Oncol 1987; 9 (1): 1–11 3602425 10.1016/s0167-8140(87)80213-x

[4] Rothwell R I, Kelly S A, Joslin C A Radiation pneumonitis in patients treated for breast cancer. Radiother Oncol 1985; 4 (1): 9–14 3929337 10.1016/s0167-8140(85)80056-6

[5] Marks L B, Yu X, Vujaskovic Z, Small W Jr, Folz R, Anscher MS Radiation-induced lung injury. Semin Radiat Oncol 2003; 13 (3): 333–345 12903021 10.1016/S1053-4296(03)00034-1

[6] Kahan Z, Csenki M, Varga Z et al The risk of early and late lung sequelae after conformal radiotherapy in breast cancer patients. Int J Radiat Oncol Biol Phys 2007; 68 (3): 673–681 17350177 10.1016/j.ijrobp.2006.12.016

[7] Hermann T S J, Molls M Radiation Pneumopathy. Experimental and clinical data In: Dunst J, Sauer R H (eds). Late Sequelae in Oncology. Berlin, Heidelberg, New York, 1995: 135–140

[8] Lind P A, Wennberg B, Gagliardi G, Fornander T Pulmonary complications following different radiotherapy techniques for breast cancer, and the association to irradiated lung volume and dose. Breast Cancer Res Treat 2001; 68 (3): 199–210 11727957 10.1023/a:1012292019599

[9] Marks L B, Yorke E D, Jackson A et al Use of normal tissue complication probability models in the clinic. Int J Radiat Oncol Biol Phys 2010; 76 (suppl 3): S10–S19 20171502 10.1016/j.ijrobp.2009.07.1754 PMC4041542

[10] Bentzen S M, Skoczylas J Z, Overgaard M, Overgaard J Radiotherapy-related lung fibrosis enhanced by tamoxifen. J Natl Cancer Inst 1996; 88 (13): 918–922 8656444 10.1093/jnci/88.13.918

[11] Dorr W, Bertmann S, Herrmann T Radiation induced lung reactions in breast cancer therapy. Modulating factors and consequential effects. Strahlenther Onkol 2005; 181 (9): 567–573 16170483 10.1007/s00066-005-1457-9

[12] Lind P A, Bylund H, Wennberg B, Svensson C, Svane G Abnormalities on chest radiographs following radiation therapy for breast cancer. Eur Radiol 2000; 10 (3): 484–489 10757001 10.1007/s003300050081

[13] West C M, Elliott R M, Burnet N G The genomics revolution and radiotherapy. Clin Oncol (R Coll Radiol) 2007; 19 (6): 470–480 17419040 10.1016/j.clon.2007.02.016

[14] Johansson S, Bjermer L, Franzen L, Henriksson R Effects of ongoing smoking on the development of radiation-induced pneumonitis in breast cancer and oesophagus cancer patients. Radiother Oncol 1998; 49 (1): 41–47 9886696 10.1016/s0167-8140(98)00064-4

[15] Vogelius I R, Bentzen S M A literature-based meta-analysis of clinical risk factors for development of radiation induced pneumonitis. Acta Oncol 2012; 51 (8): 975–983 22950387 10.3109/0284186X.2012.718093 PMC3557496

[16] Lind P A, Rosfors S, Wennberg B, Glas U, Bevegard S, Fornander T Pulmonary function following adjuvant chemotherapy and radiotherapy for breast cancer and the issue of three-dimensional treatment planning. Radiother Oncol 1998; 49 (3): 245–254 10075257 10.1016/s0167-8140(98)00121-2

[17] Lehne G, Lote K Pulmonary toxicity of cytotoxic and immunosuppressive agents. A review. Acta Oncol 1990; 29 (2): 113–124 2185802 10.3109/02841869009126530

[18] Taghian A G, Assaad S I, Niemierko A, Floyd S R, Powell S N Is a reduction in radiation lung volume and dose necessary with paclitaxel chemotherapy for node-positive breast cancer? Int J Radiat Oncol Biol Phys 2005; 62 (2): 386–391 15890579 10.1016/j.ijrobp.2004.09.044

[19] Theuws J C, Kwa S L, Wagenaar A C et al Prediction of overall pulmonary function loss in relation to the 3-D dose distribution for patients with breast cancer and malignant lymphoma. Radiother Oncol 1998; 49 (3): 233–243 10075256 10.1016/s0167-8140(98)00117-0

[20] Azria D, Belkacemi Y, Romieu G et al Concurrent or sequential adjuvant letrozole and radiotherapy after conservative surgery for early-stage breast cancer (CO-HO-RT): a phase 2 randomised trial. Lancet Oncol 2010; 11 (3): 258–265 20138810 10.1016/S1470-2045(10)70013-9

[21] Halyard M Y, Pisansky T M, Dueck A C et al Radiotherapy and adjuvant trastuzumab in operable breast cancer: tolerability and adverse event data from the NCCTG Phase III Trial N9831. J Clin Oncol 2009; 27 (16): 2638–2644 19349549 10.1200/JCO.2008.17.9549 PMC2690390

[22] Belkacemi Y, Gligorov J, Ozsahin M et al Concurrent trastuzumab with adjuvant radiotherapy in HER2-positive breast cancer patients: acute toxicity analyses from the French multicentric study. Ann Oncol 2008; 19 (6): 1110–1116 18344537 10.1093/annonc/mdn029

[23] Breast cancer atlas for radiation therapy planning: consensus definitions. 2011. http://www.rtog.org/CoreLab/ContouringAtlases/BreastCancerAtlas.aspx Accessed on 27^th^ October 2011.

[24] Fontanilla H P, Woodward W A, Lindberg M E, Kanke J E Current clinical coverage of Radiation Therapy Oncology Group-defined target volumes for postmastectomy radation therapy. Pract Radiat Oncol 2012; 2 (3): 201–209 24674124 10.1016/j.prro.2011.10.001

[25] Erven K, Weltens C, Nackaerts K, Fieuws S, Decramer M, Lievens Y Changes in pulmonary function up to 10 years after locoregional breast irradiation. Int J Radiat Oncol Biol Phys 2012; 82 (2): 701–707 21398052 10.1016/j.ijrobp.2010.12.058

[26] Prochazka M, Granath F, Ekbom A, Shields P G, Hall P Lung cancer risks in women with previous breast cancer. Eur J Cancer 2002; 38 (11): 1520–1525 12110499 10.1016/s0959-8049(02)00089-8

